# Metabolomic Profiling of *Bradyrhizobium diazoefficiens*-Induced Root Nodules Reveals Both Host Plant-Specific and Developmental Signatures

**DOI:** 10.3390/ijms17060815

**Published:** 2016-05-27

**Authors:** Martina Lardi, Valérie Murset, Hans-Martin Fischer, Socorro Mesa, Christian H. Ahrens, Nicola Zamboni, Gabriella Pessi

**Affiliations:** 1 Department of Plant and Microbial Biology, University of Zürich, CH-8057 Zürich, Switzerland; marti.lardi@access.uzh.ch; 2 Institute of Microbiology, Eidgenössische Technische Hochschule (ETH) Zürich, CH-8093 Zürich, Switzerland; vmurset@hotmail.com (V.M.); fischeha@ethz.ch (H.-M.F.); 3 Department of Soil Microbiology and Symbiotic Systems, Estación Experimental del Zaidín, Consejo Superior de Investigaciones Científicas (CSIC), E-18080 Granada, Spain; socorro.mesa@eez.csic.es; 4 Agroscope, Institute for Plant Production Sciences, Research Group Molecular Diagnostics, Genomics and Bioinformatics & Swiss Institute of Bioinformatics (SIB), CH-8820 Wädenswil, Switzerland; christian.ahrens@agroscope.admin.ch; 5 Institute of Molecular Systems Biology, ETH Zürich, CH-8093 Zürich, Switzerland

**Keywords:** host-specific nodule metabolism, metabolomics, *nifA*, *nifH* transcriptomics, nodule development, rhizobia, symbiosis

## Abstract

*Bradyrhizobium diazoefficiens* is a nitrogen-fixing endosymbiont, which can grow inside root-nodule cells of the agriculturally important soybean and other host plants. Our previous studies described *B. diazoefficiens* host-specific global expression changes occurring during legume infection at the transcript and protein level. In order to further characterize nodule metabolism, we here determine by flow injection–time-of-flight mass spectrometry analysis the metabolome of (i) nodules and roots from four different *B. diazoefficiens* host plants; (ii) soybean nodules harvested at different time points during nodule development; and (iii) soybean nodules infected by two strains mutated in key genes for nitrogen fixation, respectively. Ribose (soybean), tartaric acid (mungbean), hydroxybutanoyloxybutanoate (siratro) and catechol (cowpea) were among the metabolites found to be specifically elevated in one of the respective host plants. While the level of C4-dicarboxylic acids decreased during soybean nodule development, we observed an accumulation of trehalose-phosphate at 21 days post infection (dpi). Moreover, nodules from non-nitrogen-fixing bacteroids (*nifA* and *nifH* mutants) showed specific metabolic alterations; these were also supported by independent transcriptomics data. The alterations included signs of nitrogen limitation in both mutants, and an increased level of a phytoalexin in nodules induced by the *nifA* mutant, suggesting that the tissue of these nodules exhibits defense and stress reactions.

## 1. Introduction

*Bradyrhizobium diazoefficiens* (previously named *Bradyrhizobium japonicum*) is an α-proteobacterium able to undergo nitrogen-fixing symbiosis in determinate root nodules of several legumes including *Glycine max* (soybean), *Macroptilium atropurpureum* (siratro), *Vigna unguiculata* (cowpea) and *Vigna radiata* (mungbean) [1]. As nitrogen is a limiting nutrient in many soils, legumes have a competitive advantage over other non-legume plant families since they receive a bulk of their reduced nitrogen needs from the rhizobial partner. The establishment of a successful symbiotic interaction is coordinated by both partners and results in the formation of a root nodule structure that contains millions of intracellular, nitrogen-fixing bacteroids [2,3,4,5,6,7]. The signal exchange begins with the secretion of flavonoids by the legumes, which are recognized by the rhizobial partner that then induces expression of the *nod* genes. Nod gene products are responsible for the synthesis of lipochitooligosaccharides (Nod factors) that induce root hair curling, which helps to trap the rhizobia, and a massive subcortical cell division in the plant. Rhizobia usually enter the root hair through infection threads (IT), tubular structures formed by the plant. Once released from the IT within the cytoplasm of plant cortical cells, rhizobia are surrounded by a plant membrane called the peribacteroid or symbiosome membrane (SM) forming an organelle, in which rhizobia continue to grow and divide until infected plant cells are packed with thousands of symbiosomes [8,9]. Rhizobia within symbiosomes eventually differentiate into a nitrogen-fixing form known as the bacteroid. The continued plant and bacterial cell division leads to a mature root nodule structure that requires a constant integration of plant and bacterial metabolism to efficiently fix atmospheric nitrogen [5,10,11,12]. One essential signal for the activation of the nitrogen fixation process is a reduction of the free oxygen concentration below 25 nM within the nodule tissue [13]. The energy demands of symbiotic nitrogen fixation (16 ATP molecules to reduce one N_2_ molecule) are fuelled by photosynthetically assimilated carbon (mainly sucrose), which is metabolized through the glycolytic pathway of the host, translocated as malate or related C4-carboxylic acids across the SM and provided to bacteroids as major energy and carbon source [14]. Atmospheric nitrogen is reduced to ammonium, which is incorporated into ureides in determinate nodules, or into glutamine and asparagine in indeterminate nodules before being exported from nodules to the plant to be further metabolized [10,15,16,17].

In the past, we contributed to the elucidation of mechanisms that underlay the regulation of symbiotic nitrogen-fixation inside nodules using the rhizobial model organism *B. diazoefficiens* and a combination of transcriptomics and proteomics analyses [18,19,20,21]. These studies allowed us to (i) identify genes and proteins specifically up-regulated during soybean symbiosis compared to free-living conditions [18,19]; (ii) capture the transcriptional changes during soybean nodule development [18]; (iii) determine the importance of several nitrogen fixation regulators (RegR, NifA, RpoN, FixJ FixK_2_) by comparing transcription profiles of nodules elicited by respective mutant strains with that of wild-type induced nodules [18,22,23]; and (iv) elucidate the molecular mechanisms underlying the adaptation of *B. diazoefficiens* to different host plants [20]. One important aspect that has been missing from these studies so far was a comprehensive metabolite analysis of *B. diazoefficiens*-induced nodules. Such data are expected to provide an additional level of information by revealing plant and bacterial physiological adaptations specifically induced within root nodules. Previous studies have indeed only analyzed the metabolome of soybean roots and root hairs in response to *B. diazoefficiens* infection [24], and only more recently of *B. diazoefficiens* free-living bacteria and differentiated bacteroids from soybean [25].

In this study, we compare the metabolite profile of *B. diazoefficiens*-induced nodules in four different host plants with that of uninfected roots. This allowed us to identify metabolites that exhibited a substantial increase in the nodules of the respective host plants. Apart from C4-dicarboxylic acids, we found several amino acids, ureides, sugars like sucrose and glucose as well as (several) marker metabolites whose abundances were specifically increased in a given host plant. In addition to the metabolite profile of soybean nodules from different developmental stages (13, 21 and 31 days post inoculation (dpi)), two bacterial mutant strains known to induce Fix^−^ nodules were studied to explore the response of the legume to non-effective bacteroids. The integration of transcriptome and metabolome datasets with nodules induced by *nifH* and *nifA* mutant strains helped us to dissect plant from bacteroid metabolism. The data clearly showed that in absence of *nifH*, bacteroids are nitrogen starved but the plant is still providing photosynthates. In contrast, in absence of the NifA regulator, root nodules showed a drastically reduced level of C4-dicarboxylic acids and an accumulation of compounds usually involved in defense-like reactions.

## 2. Results and Discussion

### 2.1. Metabolomic Analysis of B. diazoefficiens Root Nodules Identifies a Core Nodule Metabolome

Metabolites were extracted from soybean, cowpea and mungbean root nodules infected by *B. diazoefficiens* at 21 dpi, and from siratro nodules at 31dpi (Table 1). These time points were chosen based on prior results revealing that respective root nodules showed maximal nitrogen fixation activity at this stage [20]. To establish a metabolic host plant baseline, we analyzed in parallel uninfected root material from each host plant (Table 1). All samples were prepared in triplicates (independent biological replicates) and each replicate was injected twice (see Materials and Methods Section). Non-targeted metabolomics by flow injection–time-of-flight mass spectrometry [26,27] was used to analyze the extracts, allowing to detect ions originating from metabolites of central metabolism. These ions were putatively annotated based on the accurate mass (tolerance 0.001 Da). After filtering of unknowns, low-abundance signals, heavy isotopes, and adducts (see Materials and Methods Section), a total of 223 ions could be matched to deprotonated metabolites (supplementary materials Table S1). Notably, this procedure does not allow distinguishing compounds with the same molecular formula or weight (e.g., sucrose, cellobiose, maltose, and trehalose). Hierarchical clustering analysis (HCA) of the metabolites detected in the different host plants infected with wild-type *B. diazoefficiens* demonstrated a high reproducibility of the experiments since all replicates of one condition build strong clusters with very similar profiles (supplementary materials Figure S1). In order to identify nodule-specific compounds, we first compared the metabolite levels measured in all nodules samples to those measured in all root samples (*i.e.*, from uninoculated plants). Out of all detected metabolite ions, we found 153 to be significantly different between nodules and root samples regardless of the host (increase/decrease of more than 1.4 (log_2_0.5)-fold in the nodule samples compared to root samples, *q*-value ≤ 0.01; see Materials and Methods Section). Among these, 132 showed a statistically significant increased amount in nodules while 21 showed increased level in the roots (supplementary materials Table S2).

Succinyl-homoserine was the most highly accumulated metabolite in all nodules compared to all roots (supplementary materials Table S2). Succinyl-homoserine is a substrate for MetZ, an enzyme required for methionine biosynthesis, which has been shown in *Rhizobium etli* to be essential for nodulation of *Phaseolus vulgaris* [28].

The C4-dicarboxylate compounds succinate, malate and fumarate that are produced by the plant and used as carbon source by the bacteroids to fuel the process of nitrogen fixation [14] are present at a higher level in all nodules samples compared to the respective root samples (supplementary materials Table S2). Further metabolites present in increased amounts in all *B. diazoefficiens*-induced nodules included the amino acids glutamate, glutamine, proline, serine and glycine. The accumulation of these amino acids has also been reported in nodules induced by *Mesorhizobium loti* in *Lotus japonicus* and in *Sinorhizobium meliloti*-elicited *Medicago sativa* nodules [29,30,31]. In a previous metabolomics approach [25], glutamate was exclusively found in soybean bacteroids, but not in free-living bacteria. Moreover, glutamate has been previously proposed to be a respiratory substrate of bacteroids [32]. Asparagine, a major export form of combined nitrogen from indeterminate nodules, showed increased levels in all *B. diazoefficiens*-induced nodules that however, was not statistically significant. Higher levels of the ureide allantoin, which is the major nitrogenous substance transported in the xylem of tropical plants such as soybean and cowpea [15,17,33,34,35] were found in all *B. diazoefficiens*-induced nodules compared to uninfected roots. The polyol glycerol, as well as sugars such as hexoses, pentose-P, and disaccharides (C12H22O11), were more abundant in nodules compared to the roots. The accumulation of glycerol-3-P and glycerone in nodules is in line with our previous transcriptomics studies where we could show that the glycerol-3-P dehydrogenase (Blr2436) is up-regulated in bacteroids compared to free-living bacteria [18]. The nucleosides adenosine, guanosine and uridine and their respective nucleotides adenosine monophosphate (AMP), guanosine monophosphate (GMP) and uridine monophosphate (UMP) are more abundant in presence of the symbiont confirming previous results that nucleotide and nucleoside metabolism is active in nodules [19,25]. Moreover, purines have been reported to serve as precursors of ureides in several plants [36,37]. An accumulation of adenosine and other purines and pyrimidines has already been described in a previous metabolite analysis on *M. sativa* nodules [30].

Among the 21 metabolites significantly more abundant in all tested roots compared to the nodules we found the phytoalexin resveratrol, urea-carboxylate, methenyltetrahydrofolate (a precursor of folate biosynthesis), hydroxypyruvate involved in glyoxylate and dicarboxylate metabolism, the nicotinamide adenine dinucleotide (NAD) precursor nicotinate D-ribonucleotide and the vitamin B1 derivative thiamin monophosphate.

In summary, by comparing the metabolic profiles of nodules samples coming from different host plants with the corresponding samples originating from uninfected roots we were able to identify a core nodule metabolome, which contains a number of metabolites possibly important for symbiosis.

### 2.2. Host-Specific Nodule and Root Metabolome

**Figure 1:**
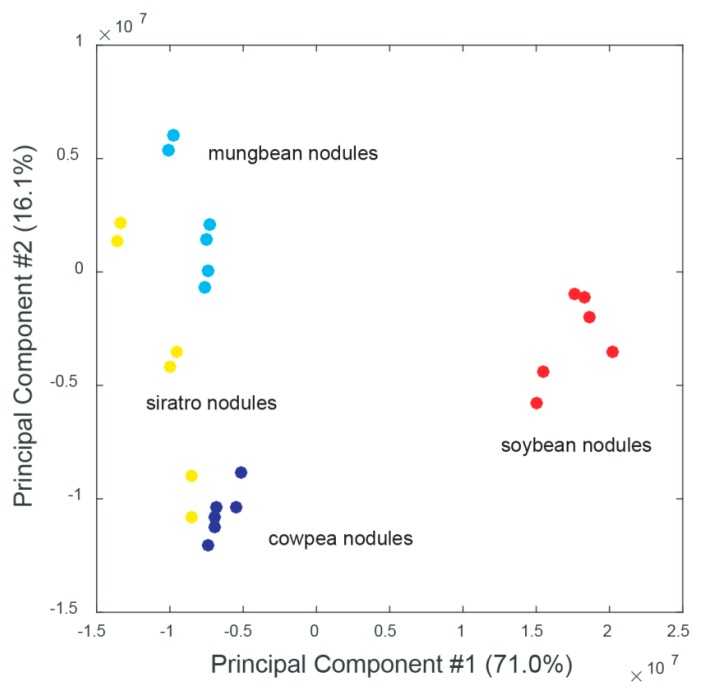
Principal component analysis (PCA) of metabolome datasets obtained from soybean (red); mungbean (light blue); cowpea (blue) and siratro (yellow) root nodules. Three biological replicates were examined, each analyzed twice by non-targeted metabolomics; #: number.

**Figure 2:**
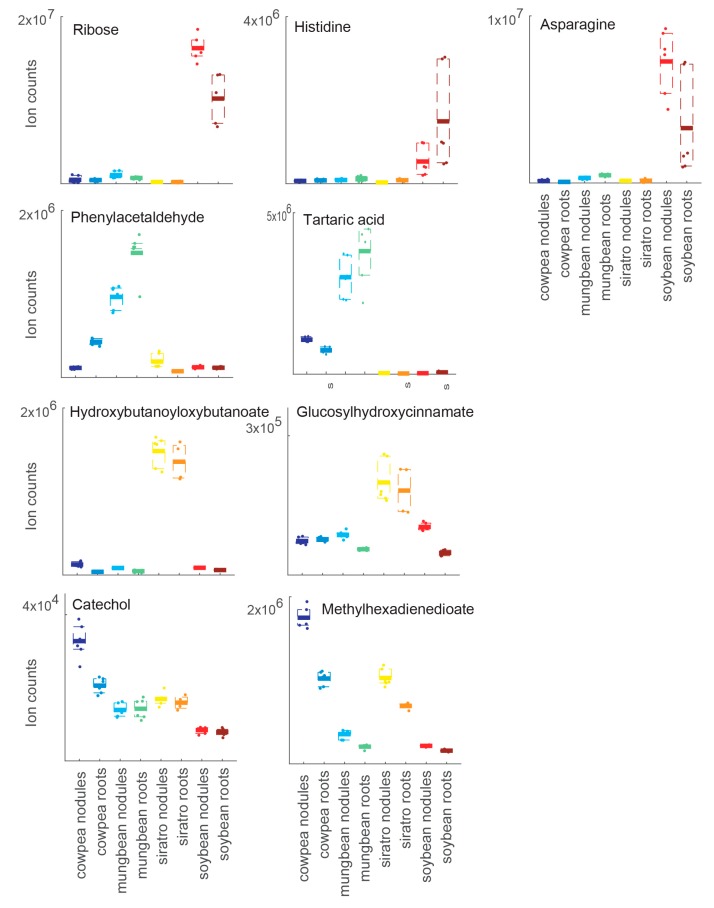
Ion counts of host-specific metabolic markers such as sucrose, asparagine, pentose (ribose) and histidine for soybean, tartaric acid and phenylacetaldehyde for mungbean, hydroxybutanoyloxybutanoate and glucosylhydroxycinnamate for siratro, and methylhexadienedioate and catechol for cowpea nodules and roots. Three biological replicates were examined, each analyzed twice by non-targeted metabolomics.

To investigate potential host-specific adaptations of *B. diazoefficiens* to different host plants at the metabolic level, the metabolite profiles of soybean, cowpea, mungbean and siratro nodules were mutually compared. We previously observed that *B. diazoefficiens* exhibits comparable nitrogenase activity during symbiosis with all four plants (our unpublished results). Principal component analyses (PCAs) was able to separate the biological samples according to their plant host. While cowpea, mungbean and siratro nodule replicates clustered more closely together, the metabolite content of the soybean nodules showed substantial differences (Figure 1, supplementary materials Figure S2). A similar clustering was observed when the metabolome of uninfected roots from the different host plants was compared by PCA analysis (supplementary materials Figure S2). Interestingly, for each host plant, a cluster of specifically accumulated metabolites could be detected: pentose (ribose), asparagine and histidine for soybean, tartaric acid and phenylacetaldehyde for mungbean, hydroxybutanoyloxybutanoate and glucosyl-hydroxycinnamate for siratro, and methylhexadienedioate and catechol for cowpea (Figure 2, supplementary materials Table S3). A comparative analysis showed that the highest number of specifically accumulated metabolites (67) was observed in the soybean host (Table 1; supplementary materials Table S3). This observation is supported by our previous transcriptome and proteome studies which had shown that *B. diazoefficiens* displays a higher number of host-specific transcripts/proteins in soybean nodules compared to nodules from the other host plants [20].

For example, dihydrouracil was found to accumulate 20-fold in soybean nodules compared to other plant nodules; accordingly, the enzyme d-hydantoinase (Blr3295), which converts 5,6-dihydrouracil into 3-ureido-propionate was detected only in siratro and cowpea bacteroids in our previous transcriptomic and proteomic expression study [20]. The amino acids asparagine, histidine, valine, leucine, threonine, glycine, serine and phenylalanine as well as the ureide allantoin and its derivative *S*-ureidoglycine are more abundant in soybean nodules compared to other nodules (supplementary materials Table S3) suggesting a higher N content in soybean nodules. In our previous study of host-specific gene and/or protein expression [20], bll7236, which encodes a threonine synthase, was shown to be up-regulated in soybean nodules compared to other nodules suggesting that the accumulated threonine in soybean nodules could be synthetized by the bacteria. Further support for this hypothesis is provided by the fact that threonine is not accumulating in soybean roots compared to other roots. Interestingly, proline only accumulated in soybean roots and one of the genes contributing to the conversion of proline into ornithine (bll2855) has been previously shown to be specifically up-regulated in soybean nodules compared to nodules of the other plants [20]. Ribose, glucose and glycerone showed increased levels in soybean roots and nodules suggesting that these C-sources could be mainly used during soybean symbiosis. In accordance with 3-hydroxybutanoate accumulation in soybean roots and nodules (supplementary materials Table S3), our previous transcriptomics study had shown that the gene encoding a poly-hydroxyalkanoate (PHA) depolymerase (blr0899), which is depolymerizing PHA to 3-hydroxybutanoate was up-regulated only in soybean nodules compared to other nodules [20].

Among the 30 metabolites specifically accumulating in mungbean nodules and/or mungbean roots (Table 1; supplementary materials Table S3), we found tartaric acid to be the most highly accumulated metabolite in mungbean compared to the other host plants. In mungbean roots and nodules, there is a striking accumulation of aromatic compounds such as phenylacetaldehyde, cinnamate, naphthalene-diol, anthranilate, phenol, dihydroxybenzoate and toluate compared to the other host plants. In mungbean nodules, we also observed the accumulation of three compounds of the tricarboxylic acid (TCA) cycle (the two tricarboxylic acids citrate and aconitate as well as the dicarboxylic acid fumarate) (supplementary materials Table S3).

By inspecting the 17 metabolites specifically accumulating in the siratro host, we found nicotinate ribonucleotide specifically accumulating in siratro roots and uridine triphosphate (UTP) and uridine diphosphate (UDP) as well as shikimate showing higher amounts in siratro nodules (Table 1; supplementary materials Table S3). The metabolites hydroxybutanoyloxybutanoate and glucosylhydroxycinnamate specifically accumulate in siratro nodules and roots (Figure 2).

Among the 17 compounds showing significantly increased amounts in cowpea nodules and/or roots compared to the other plants, we found methylhexadienedioate and catechol, which could be used to transport iron (Figure 2) and the nucleotide sugar GDP-fucose (Table 1; supplementary materials Table S3).

By comparing the metabolite profile of nodules and roots from the four host plants, we were thus able to identify host-specific alterations that could form a basis to explain differences in the metabolism of *B. diazoefficiens* in symbiosis with different host plants.

### 2.3. Metabolite Profiling during Different Stages of Soybean Nodule Development

**Figure 3:**
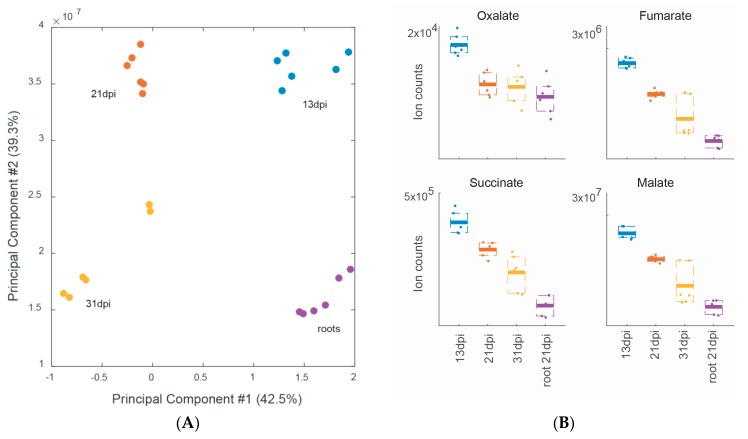
PCA of soybean wild-type nodules at different developmental stages: 13 dpi (blue), 21 dpi (red) and 31 dpi (yellow); root only material (purple); #: number (**A**); Three biological replicates were examined, each analyzed twice by non-targeted metabolomics; Differential accumulation of oxalate and C4-dicarboxylates (**B**).

The comparison between the metabolic profile of soybean nodules collected early and late in bacteroid and nodule development (13, 21 and 31 dpi) revealed important metabolic changes (Figure 3A,B; supplementary materials Figure S3), some of which are in line with the requirement of the bacteroids for C4-dicarboxylic acids to satisfy their high energy requirements to fix nitrogen at a maximal rate at 21 dpi. We indeed observed that the amount of malate, fumarate and succinate is maximal at 13 dpi and decreases over time suggesting increased carbon and energy demand of multiplying and nitrogen-fixing bacteroids (Figure 3B). The C2 compounds oxalate and glyoxylate and tartaric acid as well as ribose-5-P also showed a similar decreasing profile during soybean nodule development. In Table 2, we list the 11 metabolites that exhibited differential abundance during soybean nodule development. Among the 6 metabolites significantly more abundant at 13 dpi, we found fumarate and oxalate, phospho*enol*pyruvate, tryptophan, cyclohexylformamide and glutamyltaurine (Table 2). We know from our previous study that *B. diazoefficiens* is able to metabolize oxalate in free-living growth conditions [38], and that the enzymes responsible for the oxidation of oxalate to formate and CO_2_, the formyl-CoA transferase Frc (Bll3156) and the oxalyl-CoA decarboxylase Oxc (Bll3157) were expressed at the gene and protein level in 21 dpi old nodules [19], suggesting that oxalate may be used inside soybean nodules. In contrast, the only metabolite that accumulates specifically at 21 dpi was the disaccharide trehalose-6-phosphate (trehalose-6-P), a precursor of trehalose which has been shown previously to be produced and stored by *Bradyrhizobium* to survive during nodule senescence, oxidative stress and desiccation [39,40,41,42,43]. Notably, a previous metabolomics study on *B. diazoefficiens* showed a 92% increase of trehalose in bacteroids compared to free-living bacteria [25]. Moreover, Brechenmacher *et al.* (2010) [24] showed that a gene coding for a trehalose phosphatase from soybean was up-regulated in root hairs infected by *B. diazoefficiens*. At 31 dpi when bacteroids show reduced nitrogen fixation [18] and start to enter senescence in our experimental system, four metabolites showed significantly elevated amounts compared to the other developmental stages: glucosamine-P (a carbohydrate component of bacterial and plant cell wall polysaccharides), indole acetate, isopropylmaleate and AMP (Table 2).

Taken together, these analyses allowed us to identify metabolites with significantly increased abundance at a specific stage of soybean nodule development, which could possibly be used as developmental markers.

### 2.4. Metabolomic Analysis of a B. diazoefficiens nifA and nifH Mutant

**Figure 4:**
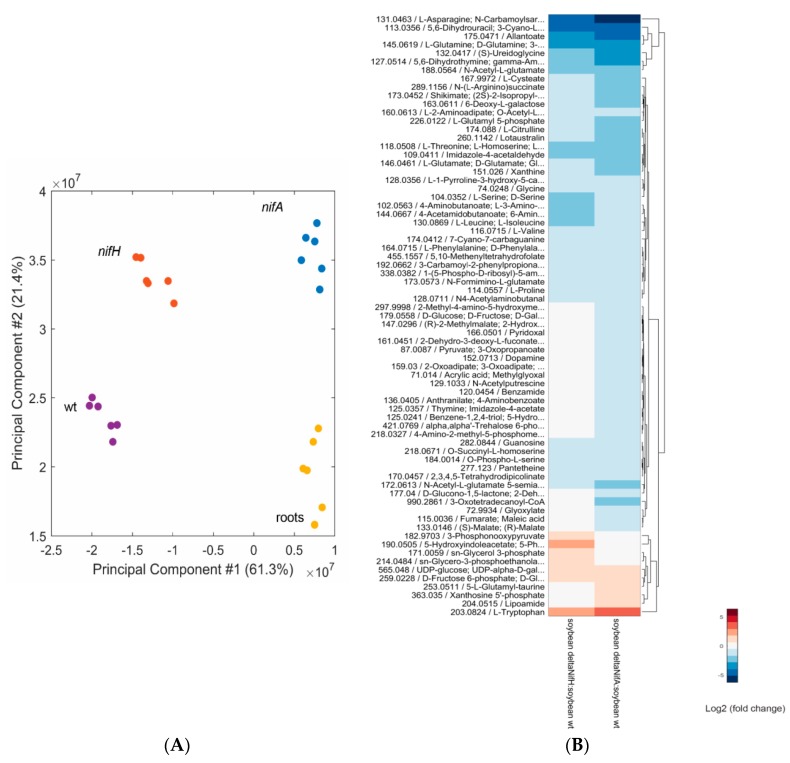
PCA of metabolite profiles from soybean wild-type nodules (purple), *nifA* nodules (blue), *nifH* nodules (orange) and soybean root only material (yellow) metabolite profiles; #: number (**A**); Three biological replicates were examined, each analyzed twice by non-targeted metabolomics. Significantly different metabolites (abs[log_2_(fold-change)] > 0.5 and *q*-value < 0.01 in at least one comparison); Metabolite ions were clustered for purposes of visualization (**B**).

By exploring the metabolite profile of plants nodulated by two mutant strains known to result in an ineffective Fix^−^ symbiotic interaction, we hoped to better differentiate plant and bacterial metabolism. To further strengthen hypotheses based on the metabolomics data, which cannot distinguish between plant and bacterial metabolites, we complemented the metabolome analyses of these mutant nodules by transcriptome analysis of the respective mutant bacteroids (see Materials and Methods Section). The first analyzed mutant contains a transposon Tn5 insertion in the *nifH* gene encoding the nitrogenase reductase enzyme, which, when inoculated on soybean seedlings, results in nitrogen-starved plants [44]. The second strain harbors a partial deletion in the gene encoding a regulator essential for nitrogen fixation (NifA) and additionally leads to a premature bacteroid degradation resulting in a necrotic nodule appearance [45,46,47]. A PCA analysis separated the metabolome of the nodules induced by the three strains (wild type, *nifH* and *nifA* mutants) and showed that *nifA* nodules were very different compared to wild-type nodules (Figure 4A; supplementary materials Figure S4) with 137 metabolites being differentially accumulated compared to wild-type nodules (Table 1). Interestingly, the metabolic profile of nodules induced by the non-fixing *nifA* mutant clustered closer to that of the root metabolome. In *nifH* nodules, which are also unable to produce ammonium and provide it to the host plant, the metabolic profile was closer to that of the wild type with 88 metabolites being differentially abundant (Table 1). Of the metabolites showing differential abundance 67 overlapped in both mutants, with 60 being more abundant in wild-type nodules and 7 more abundant in mutant nodules (supplementary materials Table S4). In both mutants, the abundance of a series of common metabolites such as the majority of the detected amino acids and shikimate, the precursor of aromatic amino acids, were reduced compared to the levels detected in wild-type nodules suggesting that the nitrogen supply to the host plant is impaired and that the nodules are undergoing nitrogen-starvation. To better understand which changes are contributed by the bacteroids, we also generated transcriptome data with nodules induced by the *nifH* and the *nifA* mutant. As expected, the nitrogen-limitation status is also reflected in the respective *B. diazoefficiens* bacteroid transcriptome data where we observed an activation of the bacterial nitrogen stress response (Ntr) with two P-II proteins encoding genes (*glnB* and *glnK*) being strongly up-regulated in nodules infected by a *nifH* mutant (supplementary materials Table S5). Expression of the two-component response regulator gene *ntrC* was elevated in *nifH* and *nifA* nodules (supplementary materials Tables S5 and S6). Interestingly, in the *nifH* mutant, expression of the gene cluster blr2803–blr2809 and gene *nirA* (bll4571), encoding an assimilatory nitrite reductase, which is also involved in nitric oxide (NO) detoxification [48], as well as *nirK*, encoding the respiratory nitrite reductase, was significantly induced. This may indicate that NO levels are elevated in non-fixing nodules induced by the *nifH^−^* mutant. In this regard, it is worth mentioning that a basal level of NO is present at different steps of the symbiotic interaction and in fact required for an optimal establishment of symbiosis (reviewed in [49]). In the case of the *S. meliloti–M. truncatula* interaction, NO is formed by a not completely understood mechanism, which is independent of nitrogen fixation [50]. Although the source of NO production in *B. diazoefficiens*-induced soybean nodules remains unclear when nitrate is absent, it seems that nitrogen fixation is somehow involved in controlling the proper levels of NO for an effective symbiosis. As expected, the amount of several ureides and its purine precursors was also reduced in *nifH* and *nifA* nodules (supplementary materials Table S4) likely because no ammonium is produced by these bacteroids, and, as a consequence, synthesis and transport of ureides is impaired. Furthermore, in non-fixing nodules of the *nifA* and *nifH* mutants we found elevated level of the amino acid tryptophan, which is the precursor of the plant hormone auxin (Figure 4B). Moreover, elevated levels of hexose-sugars were observed in non-fixing nodules. In addition, 71 metabolites were differentially abundant only in the small and necrotic nodules formed by the *nifA* mutant (supplementary materials Table S7) compared to *nifH* nodules. The C4 organic acids fumarate and malate as well as pyruvate, glyoxylate, glycerone and trehalose-P, which specifically accumulated at 21 dpi in wild-type induced nodules (see above, Table 2) showed reduced amounts only in *nifA* (but not *nifH*) nodules. Notably, in the *nifA* mutant, the amount of malate and fumarate was reduced to the level detected in roots (data not shown) suggesting that the host plant is sanctioning inefficient bacteroids by not providing these compounds. Congruent with previous studies showing accumulation of the phytoalexin glyceollin in nodules induced by the *nifA* mutant [51], we found an elevated level of another phytoalexin (resveratrol) in *nifA* nodules further supporting the idea that soybean plants elicit a defense response against this particular ineffective mutant.

Further support to the hypothesis that the *nifA* mutant generates defense reactions in soybean nodules is provided by our transcriptomics data of *nifA* nodules (supplementary materials Table S6): among the most highly up-regulated genes in mutant nodules compared to the wild type we found clusters of genes belonging to the *tss* cluster region (bll1797–blr1843) involved in type III secretion [52]. Expression of this system (T3SS) is induced by the flavonoid genistein, and mutant strains lacking the transcriptional activator TtsI showed a delay in nodule development and host-dependent effects on nitrogenase activity [52]. In fact, T3SS effectors have been previously shown to suppress plant defense responses [53,54,55,56] against rhizobia and to trigger incompatibility with specific soybean varieties [56,57]. Additionally, blr4635 (*groEL_6_*) encoding a chaperone was up-regulated in *nifA* nodules suggesting that *nifA* mutant bacteroids are stressed inside the aberrant nodules. A list of the genes highly differentially expressed (threshold log_2_ ≥ 4 or ≤ −4) in nodules infected by the *nifA* and *nifH* mutant compared to wild-type nodules is presented in Table 3.

Overall, the comparison of the metabolite profile of soybean nodules induced by inefficient mutant bacteria with that of wild-type nodules indicated that the plant can sense the presence of efficient and inefficient rhizobia and react differentially to the presence of efficient and inefficient rhizobia by altering the level of several metabolites. Notably, the differential metabolite profile of nodules elicited by *nifH* and *nifA* mutants indicated that properties other than the lack of nitrogen fixation (which is common to both mutants) contribute to the balanced host-symbiont interaction. The combined metabolome and transcriptome data allowed us to tease apart the contribution from plant and bacterium, which otherwise is not straightforward with metabolomics data alone.

## 3. Materials and Methods

### 3.1. Bradyrhizobium Diazoefficiens Strains and Plant Growth

Seeds of soybean (*G. max* (L.) Merr. cv. Williams 82), cowpea (*V. unguiculata* (L.) Walp. cv. Red Caloona), mungbean (*V. radiata*), and siratro (*M. atropurpureum* (DC.) Urb.) were surface-sterilized as described [20]. Germination, inoculation, and growth of the plants were done as previously described [1,59]. The seeds were inoculated with *B. diazoefficiens* cultures grown in peptone-salts-yeast extract (PSY) medium supplemented with 0.1% l-arabinose [60]. *B. diazoefficiens* wild-type strain 110*spc4* [60] and mutant strains H1 [44] and A9 [45] were used. Table 1 is providing an overview of all conditions tested in this study.

### 3.2. Plant Harvesting and Metabolite Extraction

Soybean, cowpea and mungbean nodules were collected 21 dpi. Siratro nodules were collected at 31 dpi when nitrogen fixation activity was maximal. For comparison of metabolite abundances, uninfected roots of soybean, cowpea, mungbean and siratro were also sampled (21 and 31 dpi, respectively). Two (for siratro roots) or three (for all other root and nodule samples) biological replicates were analyzed per host plant and time point, each in two technical replicates. Approximately 20 mg nodules or roots were processed per sample. Immediately after removing a plant from soil material, all nodules from one inoculated plant or the whole root of an uninoculated plant were rapidly quenched by flash-freezing in liquid nitrogen. Unstable, short-lived metabolites may therefore potentially be lost by using this approach. Frozen nodules were crushed with a Tissuelyzer 2 times for 30 s at maximum speed (Qiagen, Valencia, CA, USA.). Two mL of cold methanol 80% (in water) were added, and the samples were kept at −20 °C for 20 min with regular vortexing. The samples were flash-frozen in liquid nitrogen and stored at −80 °C. The methanol extracts were analyzed by non-targeted flow injection—time-of-flight mass spectrometry on an Agilent 6550 QTOF instrument in negative mode ionization as described by Fuhrer and colleagues [26].

### 3.3. Data Analysis

Ions were annotated based on their accurate mass and the KEGG *Glycine max* and *B.diazoefficiens* metabolite lists (Version June 2015) [61] allowing a tolerance of 0.001 Da [26]. Unknown and low-abundance features, as well as ions adducts or heavy isotopologues were discarded. Eventually, 223 ions with distinct *m*/*z* were matched to 425 deprotonated metabolites and retained for statistical analysis. Ion intensities for each sample are available in supplementary materials Table S8. The larger number of candidate molecules compared to ions is caused by the existence of metabolites with identical molecular formula and weight. For comparative univariate analysis, we used a two-tailed, heteroscedasic *t*-test and applied false discovery rate (FDR) correction according to Storey *et al.* [62]. Changes with abs[log_2_(fold-change)] ≥ 0.5 and *q*-value ≤ 0.01 were considered significant. To identify nodule-specific compounds, the metabolite levels measured in all nodules samples (soybean, mungbean, cowpea and siratro) were compared with the level in all root samples (soybean, mungbean, cowpea and siratro).

### 3.4. Transcriptome Analysis

RNA from nodules (induced by wild-type, *nifH* and *nifA* mutant *B. diazoefficiens* strains 110*spc*4, H1, and A9, respectively) was extracted and processed as described previously [18]. Three independent replicas were processed for each strain. For cDNA preparation and labelling as well as for cDNA hybridization on the custom *B. diazoefficiens* Affymetrix GeneChip (BJAPETHa520090), the protocol described by Hauser and colleagues [21] was used. Data analysis was performed using GeneSpring GX 7.3.1 software (Agilent technologies, Palo Alto, CA, USA). After filtering on flags (present or marginal in at least two out of three replicas), a statistical student *t*-test with a *p* value threshold of 0.01 was applied. Genes were considered as differentially expressed if the abs[log_2_(fold-change)] ≥ 1 when comparing two strains. The raw data files for the two mutant strains are accessible through the Gene Expression Omnibus (GEO) Series accession number GSE79811. For comparisons wild-type nodules data (GSM210242–GSM210245) were used.

## 4. Conclusions

The comparison of the relative metabolite abundance in *B. diazoefficiens*-induced nodules *versus* uninfected roots allowed us to identify several characteristic metabolites specific for nodules (Table S2). In addition to the well-known dicarboxylates malate, succinate and fumarate other metabolites such as succinyl-homoserine, glutamine and glutamate were found. Contrary to a metabolic analysis performed with nodules formed in the *Medicago sativa*–*S. meliloti* symbiosis, which had reported that all analyzed amino acids were more abundant in nodules [30], in our study only the amino acids glutamate, glutamine, proline, serine and glycine showed significantly increased levels in the nodule tissues. Indeed, glutamate was previously found as the only differentially accumulated amino acid in the metabolite profile of *B. diazoefficiens–*soybeanbacteroids [25]. The increased amounts of several osmoprotectants (proline, trehalose and glycerol) in all nodules compared to all roots suggest that, in concordance to what has been observed in *Lotus japonicus* nodules [29,31], soybean nodules are osmotically stressed. In the same line, a prior metabolomics study on *B. diazoefficiens* showed that the abundance of trehalose increased about 92% in mature bacteroids in comparison to free-living bacteria [25]. Interestingly, our metabolite profiling over different stages of soybean nodule development also identified trehalose-6-P as the only metabolite, which specifically accumulated at 21 dpi (Table 2).

The concept of rhizobial adaptation to different plant hosts, which was investigated previously at the transcriptome and proteome level [20], was studied here at the metabolic level by analyzing *B. diazoefficiens*-induced nodules from four different legumes. Interestingly, we identify marker metabolites for each plant (Figure 2) and found that some of the *B. diazoefficiens* changes observed at the transcript and protein level were supporting abundance changes in the metabolism data, suggesting that bacteria also contribute to the observed differences in the metabolic profile [38]. The integration of metabolomics with transcriptomics and proteomics data on *B. diazoefficiens*–legume symbiosis can therefore be used to discriminate between alterations originating from the plant or bacterial partner, which is otherwise only possible using differential metabolic labeling strategies. In the context of nodule expression studies, proteomics data is of particular value as the peptide information content [63] can be used to unambiguously map back peptides to bacterial and plant proteins [64], thereby providing an important advantage over microarray studies that rely on RNA extracted from both bacteria and plants and that are prone to cross-hybridization. Particularly promising for future integrative studies of plant and symbiont are combined RNA-Seq and shotgun proteomics studies, which have recently been shown—when applying adequate strategies—to enable description of complete condition-specific expressed proteomes [65,66].

Another promising approach to dissect nodule metabolism into the respective bacterial and plant contribution is the use of nodules induced by *B. diazoefficiens* mutants defective in nitrogen fixation. While we observed clear symptoms of nitrogen (but not carbon) starvation in *nifH* nodules (confirmed also by transcript profiling), *nifA*-induced soybean nodules showed signs of additional stress: they produced more phytoalexins and the bacteroids displayed elevated expression of genes involved in type III secretion (Table 3). Indeed, it was previously shown that NifA also controls bacteroid persistence in infected soybean nodule cells [45,47].

We believe that the wealth of data presented here, including the analysis of defined symbiotic mutants, as well as data covering metabolic differences for symbiosis with four different host plants of *B. diazoefficiens*, will represent a very useful resource for the *Rhizobium* community and will stimulate further research of nodule metabolism.

## Figures and Tables

**Table 1:** Overview of experiments and summary of comparative non-targeted metabolome analyses performed in this study using flow injection time-of-flight mass spectrometry.

Strain ^a^	Host Plant	Number of Biological Replicates			dpi ^a^		
WT	soybean	3			13		
WT	soybean	3			21		
WT	soybean	3			31		
A9 (*nifA* mutant)	soybean	3			21		
H1 (*nifH* mutant)	soybean	3			21		
none (uninfected roots)	soybean	3			21		
WT	cowpea	3			21		
none (uninfected roots)	cowpea	3			21		
WT	mungbean	3			21		
none (uninfected roots)	mungbean	3			21		
WT	siratro	3			31		
none (uninfected roots)	siratro	2			31		
**Experimental Comparison ^a^**			**# incr met ^b^**	**# decr met ^c^**			**Table**
*2.2. Host-Specific Nodule and Root Metabolome (4 host plants)*							
WT *vs.* none (uninfected roots)			132	21		Table S2	
WT or none (soybean) *vs.* WT or none (cowpea, mungbean, siratro)			67	nd		Table S3	
WT or none (mungbean) *vs.* WT or none (cowpea, soybean, siratro)			30	nd		Table S3	
WT or none (siratro) *vs.* WT or none (cowpea, mungbean, soybean)			17	nd		Table S3	
WT or none (cowpea) *vs.* WT or none (mungbean, soybean, siratro)			17	nd		Table S3	
*2.3. Metabolome of different stages of nodule development (soybean)*							
WT 13 dpi *vs.* 21 and 31 dpi			6	nd		Table 2	
WT 21 dpi *vs.* 13 and 31 dpi			1	nd		Table 2	
WT 31 dpi *vs.* 13 and 21 dpi			4	nd		Table 2	
*2.4. Metabolome of nodules induced by a* nifA *and* nifH *mutant (soybean)*							
WT *vs.* A9 (*nifA* mutant)			25	112			
WT *vs.* H1 (*nifH* mutant)			19	69			

^a^: WT = wild type; dpi *=* days post inoculation; ^b^: # incr met = number of metabolites showing a statistically significant increase (log_2_ ≥ 0.5, *q*-value *≤* 0.01) in the comparison*;* ^c^: # decr met = number of metabolites showing a statistically significant decrease (log_2_ ≤ 0.5, *q*-value ≤ 0.01) in the comparison. nd = not determined.

**Table 2:** Three clusters of *B. diazoefficiens* metabolites that show differential abundance during soybean nodule development (13, 21, 31 dpi).

Metabolites Specific for the Indicated Time Point ^a^	ID ^a^	log_2_ 13 *vs* 21 ^b^	log_2_ 13 *vs* 31 ^b^	log_2_ 21 *vs* 31 ^b^
**13 dpi**				
Tryptophan	C00078	2.4	2.0	nr
Cyclohexylformamide	C11519	0.9	1.2	nr
Glutamyl-taurine	C05844	0.9	0.9	nr
Oxalate	C00209	0.6	0.7	nr
**Fumarate**	**C00122**	**0.6**	**1.3**	**ns**
Phosphoenolpyruvate	C00074	0.5	0.8	nr
**21 dpi**				
Trehalose 6-phosphate	C00689	−1.2	−0.3	0.9
**31 dpi**				
**Glucosamine 6-phosphate**	**C00352**	**nr**	**−1.2**	**−0.8**
Indole-3-acetate	C00954	nr	−1.0	−0.7
**Isopropylmaleate**	**C02631**	**nr**	**−0.8**	**−0.8**
**AMP**	**C00020**	**nr**	**−0.7**	**−0.7**

^a^: Metabolite name and ID according to the Kyoto Encyclopedia of Genes and Genomes (KEGG) database; ^b^: Log_2_ fold change (FC) of metabolite level, comparing two developmental stages. nr, not regulated. ns, regulated but not significantly. Metabolites indicated in bold face significantly accumulate in nodules from all four plants *versus* all roots.

**Table 3:** List of the 137 genes that showed high differential expression in nodules infected by a *nifH* and/or a *nifA* mutant strain when wild-type nodules were used as reference (abs[log_2_(fold-change)] > 4 and *q*-value < 0.01 in at least one of the two mutant strains).

ORF No. ^a^	Description ^b^	Gene Name ^b^	log_2_(*nifA vs.* wt) ^c^	log_2_(*nifH vs.* wt) ^c^
Energy production and conversion				
bll1718	C4-dicarboxylate transport protein	*dctA*	−4.6	−2.0
bll2063	phenolhydroxylase homolog	*nrgC*	−6.2	
bll4571	putative ferredoxin—nitrite reductase		2.2	5.0
bll6940	HupC protein	*hupC*	−5.4	
bll6941	uptake hydrogenase large subunit	*hupL*	−4.1	
bll6942	uptake hydrogenase precursor	*hupA*	−6.6	
blr1721	uptake hydrogenase large subunit homolog	*hupL*	−4.2	
blr1724	HupD protein homolog		−4.3	
blr1743	nitrogenase molybdenum-iron protein alpha chain	*nifD*	−5.4	
blr1744	nitrogenase molybdenum-iron protein beta chain	*nifK*	−5.2	
blr1745	nitrogenase molybdenum-cofactor synthesis protein	*nifE*	−6.0	
blr1746	nitrogenase molybdenum-cofactor synthesis protein	*nifN*	−5.6	
blr1765	Ferredoxin	*fer2*	−4.3	
blr1773	electron transfer flavoprotein alpha chain	*fixB*	−5.0	−1.2
blr1774	Flavoprotein	*fixC*	−5.2	
blr1816	RhcN protein	*rhcN*	4.4	
blr1853	cytochrome P450 family protein		−6.0	
blr2038	electron transfer flavoprotein beta chain	*fixA*	−4.6	
blr2143	similar to cytochrome P450-family protein		−4.9	
blr3719	hypothetical protein		−3.6	−4.3
blr3722	dihydrolipoamide dehydrogenase	*lpd*	−2.8	−4.9
bsr1739	Ferredoxin	*fdxN*	−5.1	
bsr1760	ferredoxin-like protein	*frxA*	−5.9	
bsr1775	probable ferredoxin	*fixX*	−6.3	
Amino acid transport and metabolism				
blr1756	nitrogenase metalloclusters biosynthesis protein	*nifS*	−5.4	
blr1971	putative peptidase		−4.6	
blr2071	similar to inosamine-phosphate amidinotransferase		-4.9	
blr2106	l-ectoine synthase	*ectC*	−6.5	
blr2136	putative aminotransferase		−5.4	
Carbohydrate transport and metabolism				
blr1656	putative glycosyl hydrolase		4.8	
blr2581	putative D-fructose-1,6-bisphosphatase protein	*cbbF*		5.0
Coenzyme transport and metabolism				
blr1686	putative aminotransferase protein		−6.0	
blr1852	similar to pantoate—β-alanine ligase		−4.3	
Translation, ribosomal structure and biogenesis				
blr2135	hypothetical protein		−5.0	
Transcription				
bll1906	*N*-acetyltransferase NrgA homolog		−5.0	
blr1880	transcriptional regulatory protein LuxR family		−5.0	
Replication and repair				
blr8234	unknown protein		−4.7	
Cell wall/membrane/envelop biogenesis				
bll1872	hypothetical protein		−5.5	
bll1944	hypothetical protein		−5.4	
bll2085	hypothetical protein		−4.3	
Post-translational modification, protein turnover, and chaperones				
bll1777	alkyl hydroperoxide reductase	*ahpC*	−6.0	
bll2059	GroEL3 chaperonin	*groEL3*	−4.8	
bll2060	GroES3 chaperonin	*groES3*	−4.9	
blr1879	hypothetical protein		−4.4	
Inorganic ion transport and metabolism				
bll2801	probable potential formate transporter			4.2
bll4570	probable sulfite reductase (NADPH) flavoprotein		1.6	4.8
bll5736	putative thiosulfate sulfurtransferase precursor		4.0	5.8
blr1719	molybdenum transport system permease protein	*modB*	−4.3	
blr1769	dinitrogenase reductase protein	*nifH*	−5.3	−3.3
blr2803	ABC transporter nitrate-binding protein	*nrtA*	1.6	4.2
blr3278	hypothetical protein		−4.1	
blr6951	molybdenum ABC transporter Molybdate-binding protein	*modA*	−4.9	
blr7315	unknown protein		4.3	
Secondary metabolites biosynthesis, transport, and catabolism				
bll2125	probable dioxygenase		−5.0	
blr2036	Oxidoreductase	*fixR*	−4.3	
blr2108	probable peptide synthetase		−4.6	
blr2131	probable oxygenase		−6.0	
blr2133	hypothetical protein		−5.3	
blr2144	cytochrome P-450 BJ-1	*cyp112*	−4.5	
blr2145	cytochrome P-450 BJ-3	*cyp114*	−4.2	
bsr1757	nitrogen fixation protein		−5.0	
General functional prediction only				
bll1776	alkyl hydroperoxide reductase	*ahpD*	−4.2	
blr1759	FeMo cofactor biosynthesis protein	*nifB*	−5.1	
blr2041	unknown protein		−4.2	
blr2042	hypothetical protein		−4.8	
blr7556	non-heme haloperoxidase		−1.9	4.4
Function unknown				
bll1754	hypothetical protein		−4.8	
bll1767	hypothetical protein		−6.0	
bll1810	hypothetical protein		5.4	
bll1979	hypothetical protein		−5.6	
bll1980	hypothetical protein		−4.8	
bll1981	hypothetical protein		−4.9	
bll2003	unknown protein		−4.3	
bll2009	hypothetical protein		−5.6	
bll4177	hypothetical protein		5.5	
bll5738	unknown protein		4.8	6.8
bll6381	unknown protein		−4.3	−1.0
bll6552	hypothetical protein		4.5	
blr1649	unknown protein		5.3	
blr1676	hypothetical protein		4.2	
blr1704	hypothetical protein		6.1	
blr1705	unknown protein		4.6	
blr1747	iron-molibdenum cofactor processing protein	*nifX*	−4.6	
blr1748	hypothetical protein		−6.3	
blr1755	*R. etli iscN* homolog		−4.7	
blr1761	iron-sulfur cofactor synthesis protein	*nifZ*	−4.6	
blr1770	molybdenum processing protein	*nifQ*	−5.1	−4.5
blr1771	nitrogenase stabilizing-protective protein	*nifW*	−5.7	−3.9
blr1806	unknown protein		4.9	
blr1812	nodulation protein	*nolB*	5.9	
blr1814	nodulation protein	*nolU*	5.0	
blr1817	hypothetical protein		5.1	
blr1851	unknown protein		−4.7	
blr2132	unknown protein		−6.7	
blr2140	hypothetical protein		4.2	
blr2505	hypothetical protein		−4.3	
blr6172	hypothetical protein		4.7	
blr7321	hypothetical protein		4.3	
blr7327	hypothetical protein		6.0	
bsr1749	hypothetical protein		−4.4	
Intracellular trafficking, secretion, and vesicular transport				
blr1813	RhcJ protein	*rhcJ*	5.7	
blr1819	RhcR protein	*rhcR*	4.5	
bsr1820	RhcS protein	*rhcS*	4.9	
Other				
bll1634	unknown protein		−4.9	
bll1636	unknown protein		−4.4	
bll1801	hypothetical protein		4.3	
bll1804	unknown protein		5.4	
bll1846	unknown protein		4.3	
bll1858	hypothetical protein		−5.2	
bll1877	unknown protein		4.4	
bll2004	unknown protein		−4.9	
bll2067	nodulate formation efficiency C protein	*nfeC*	−4.9	
bll6154	unknown protein		3.1	4.9
blr1638	unknown protein		−5.4	
blr1650	unknown protein		4.9	
blr1726	unknown protein		−7.0	
blr1728	HupK protein homolog	*hupK*	−4.5	
blr1763	unknown protein		−6.1	
blr1850	unknown protein		−5.2	
blr1954	unknown protein		−6.7	
blr1964	putative sugar hydrolase		−5.4	
blr1992	unknown protein		−5.5	
blr2011	unknown protein		−4.9	
blr2069	unknown protein		−4.4	
blr2134	hypothetical protein		−5.1	
blr7314	unknown protein		5.6	
bsl1637	unknown protein		−5.7	
bsl1651	unknown protein		4.2	
bsl1652	unknown protein		4.8	
bsl1808	unknown protein		5.1	
bsl1857	unknown protein		−4.8	
bsl2596	unknown protein		5.0	
bsr1758	unknown protein		−5.1	
bsr1764	unknown protein		−4.4	
bsr1907	unknown protein		−4.0	
bsr2005	unknown protein		−4.4	
bsr2010	unknown protein		−6.0	

^a^: Nomenclature and gene description according to Kaneko *et al.* (2002) [58]; functional classification according to the EggNOG classification (http://eggnogdb.embl.de); ^b^: Gene name according to the EMBL-EBI database*;* ^c^: Log_2_(fold-change) of transcript level, comparing mutant with wild-type strain.

## Supplementary Materials

Supplementary materials can be found at http://www.mdpi.com/1422-0067/17/6/815/s1.
