# Supplementary material for: Metabolomic Profiling of Bradyrhizobium diazoefficiens-Induced Root Nodules Reveals Both Host Plant-Specific and Developmental Signatures
Source: Int J Mol Sci. 2016 May 27;17(6):815. doi: 10.3390/ijms17060815 (PMC4926349; doi:10.3390/ijms17060815)
Supplement: Supplementary file 1 [file ijms-17-00815-s001.zip › ijms-127696-Supplementary Information-Figures.pdf]

Supplementary Materials: Metabolomic Profiling of *Bradyrhizobium diazoefficiens*-Induced Root Nodules Reveals Both Host Plant-Specific and Developmental Signatures

Martina Lardi, Valérie Murset, Hans-Martin Fischer, Socorro Mesa, Christian H. Ahrens, Nicola Zamboni and Gabriella Pessi

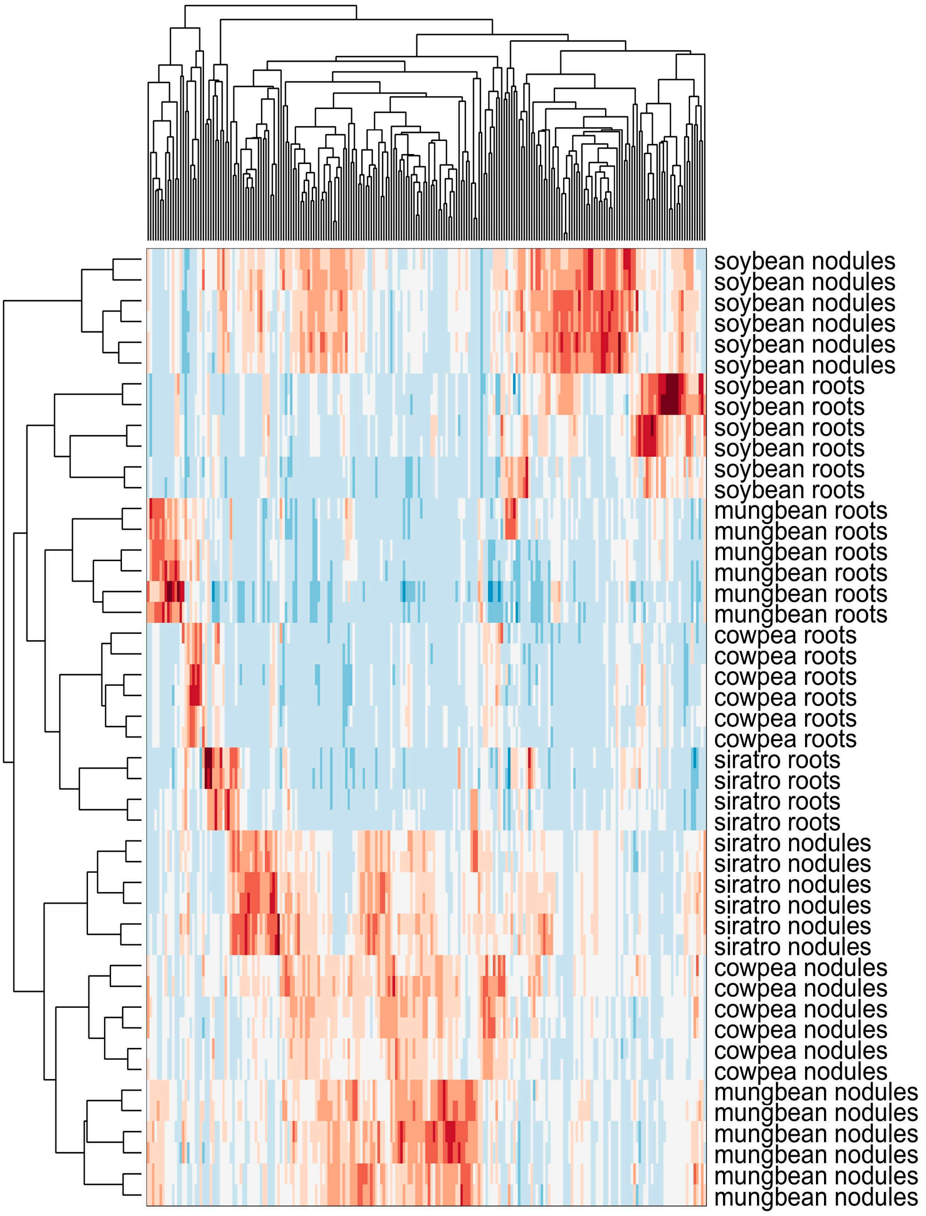

Figure S1. Hierarchical cluster analysis of all analyzed nodules and root samples. Data are *z*-score. Red = increased compared to the average, Blue = decreased compared to the average.

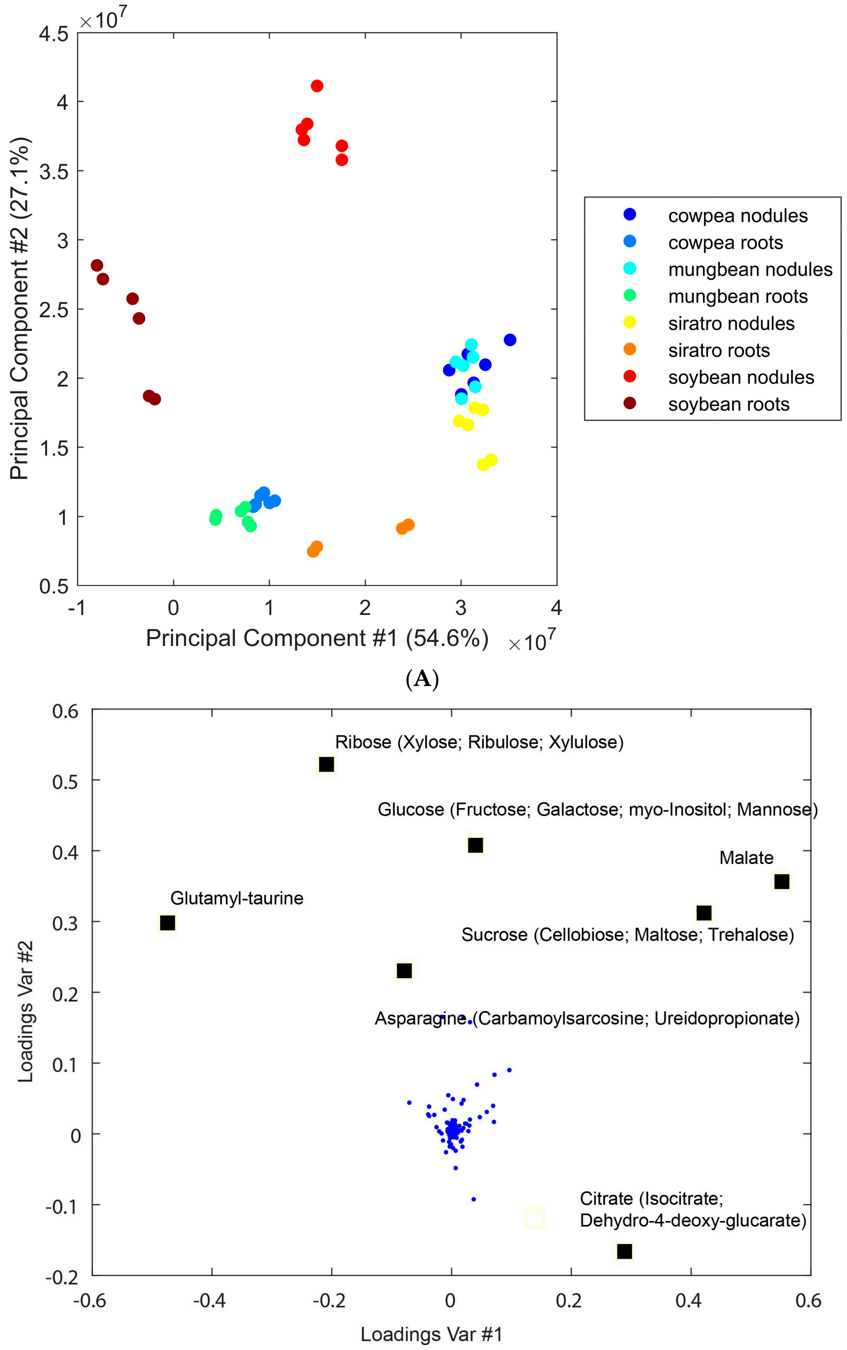

(**A**)

(**B**)

Figure S2. Principal component analysis (PCA) of soybean (red), mungbean (light blue), cowpea (blue) and siratro (yellow) nodules and its corresponding uninfected roots (**A**). Three biological replicates were examined, each analyzed twice by non-targeted metabolomics; The metabolites contributing mostly to a clear discrimination of the eight samples types in the first two principal components are visualized in the PCA loadings plot, the compounds with the same molecular formula or weight are written in parenthesis (**B**); #: number.

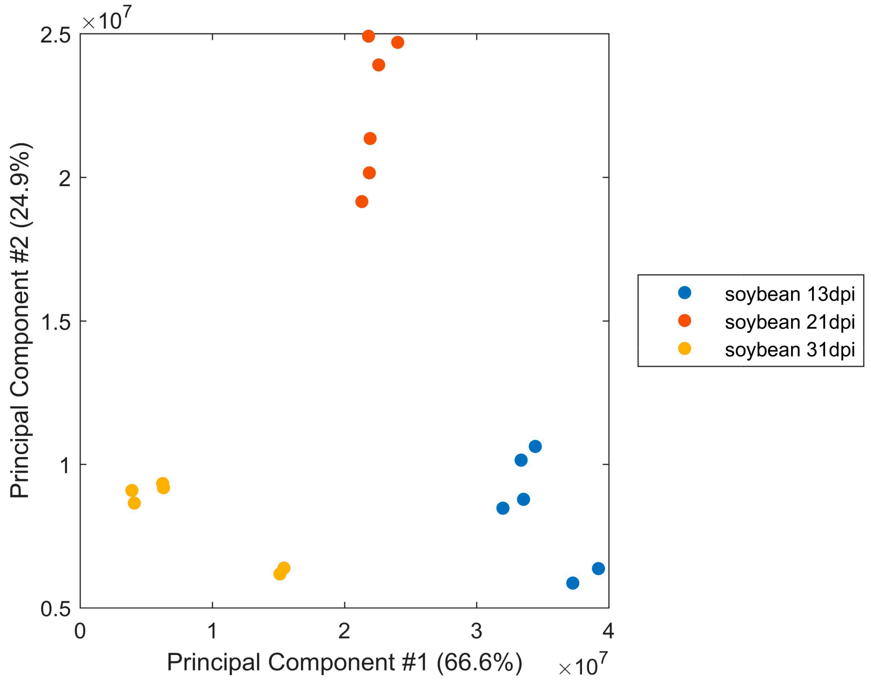

(**A**)

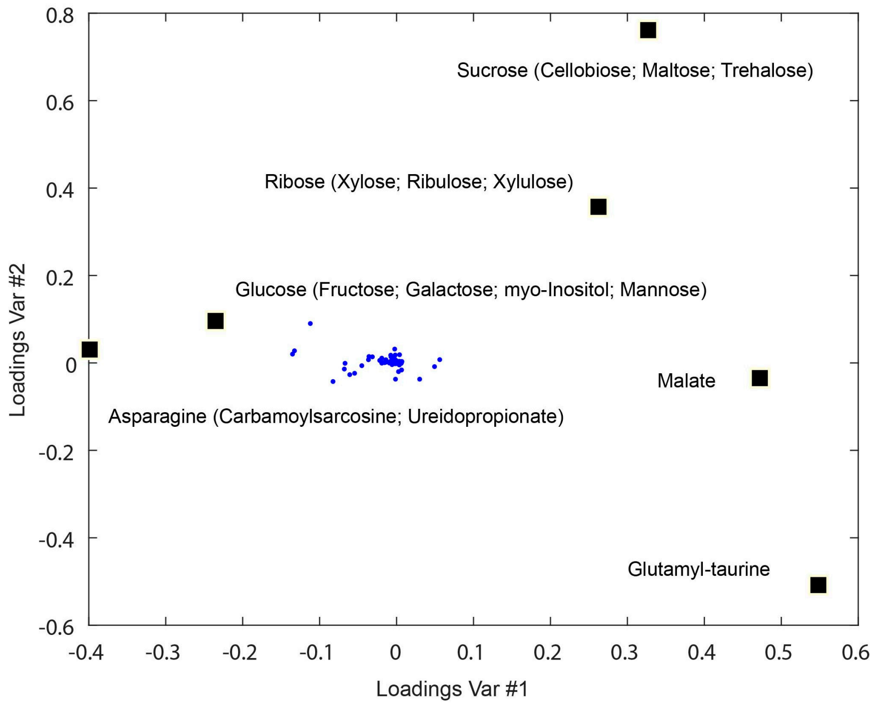

(**B**)

Figure S3. PCA of soybean wild-type nodules at different developmental stages: 13 dpi (blue), 21 dpi (red) and 31 dpi (yellow) (**A**). Three biological replicates were examined, each analysed twice by non-targeted metabolomics; The metabolites contributing mostly to a clear discrimination of the three samples types in the first two principal components are visualized in the PCA loadings plot, the compounds with the same molecular formula or weight are written in parenthesis (**B**); ; #: number

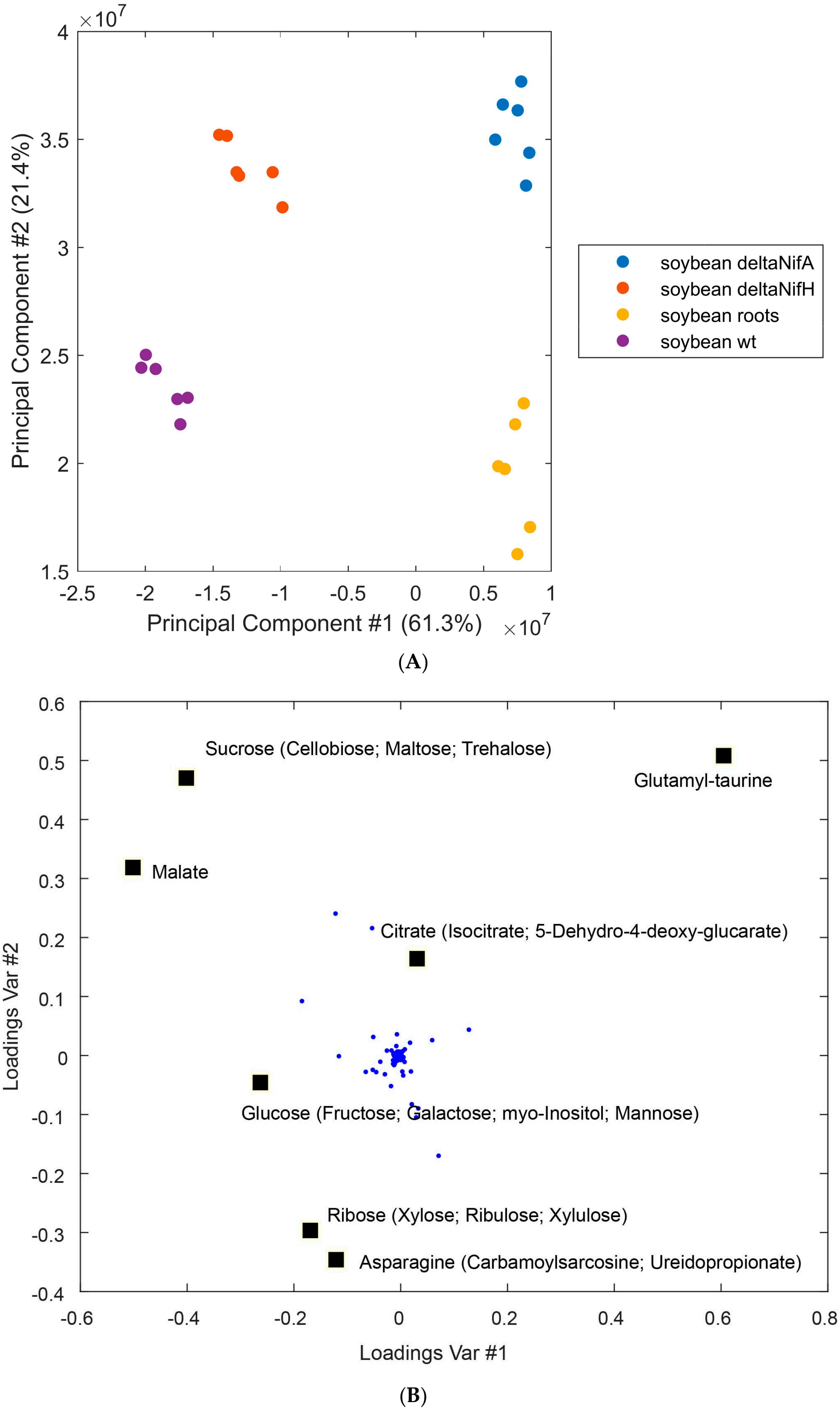

Figure S4. Principal component analysis (PCA) of soybean wild-type nodules (violet), nodules induced by a *nifH* mutant (red), nodules induced by a *nifA* mutant (blue) and soybean root only material (yellow) metabolite profiles (**A**). Three biological replicates were analyzed, each analyzed twice by non-targeted metabolomics; The metabolites contributing mostly to a clear discrimination of the three samples types in the first two principal components are visualized in the PCA loadings plot, the compounds with the same molecular formula or weight are written in parenthesis (**B**); #: number.
